# Advances and Innovations in Conjugated Polymer Fluorescent Sensors for Environmental and Biological Detection

**DOI:** 10.3390/bios15090580

**Published:** 2025-09-04

**Authors:** Viet-Duc Phung, Vinh Van Tran

**Affiliations:** 1 Institute of Fundamental and Applied Sciences, Duy Tan University, Ho Chi Minh City 70000, Vietnam; phungvietduc@duytan.edu.vn; 2 Faculty of Environmental and Chemical Engineering, Duy Tan University, Da Nang 55000, Vietnam; 3 Department of Mechanical Engineering, Gachon University, Seongnam 13120, Republic of Korea

**Keywords:** conjugated polymers, fluorescent materials, fluorescent sensors, environmental monitoring, pathogen detection

## Abstract

Thanks to their multiple outstanding features—such as high fluorescence quantum yield, good photostability, and excellent sensitivity—conjugated polymers (CPs) have emerged as a pioneering class of fluorescent materials for sensing applications, particularly in environmental and biological fields, for the detection of a wide range of environmental pollutants and bioactive compounds. The presence of delocalized π-electrons in the CP backbone significantly enhances sensing performance through a unique phenomenon known as the “molecular wire effect.” As a result, CP-based fluorescent sensors have been extensively developed and employed as exceptional tools for monitoring various analytes in environmental and biological contexts. A deep understanding of their unique properties, fabrication techniques, and recent innovations is essential for guiding the strategic development of advanced CP-based fluorescent sensors, particularly for future point-of-care applications. This study presents a critical review of the key characteristics of fluorescent sensors and highlights several common types of conjugated polymers (CPs) used in their design and fabrication. It summarizes and discusses the main sensing mechanisms, state-of-the-art applications, and recent innovations of CP-based fluorescent sensors for detecting target compounds in environmental and biological fields. Furthermore, potential strategies and future perspectives for designing and developing high-performance CP-based fluorescent sensors are emphasized. By consolidating current scientific evidence, this review aims to support the advancement of highly sensitive fluorescent sensors based on various CP nanoparticles for environmental and biological applications.

## 1. Introduction

Fluorescent sensors are among the most important tools in biological research due to their high efficiency and ability to monitor and identify target analytes or chemical reactions of interest through fluorescence signals [1]. The global fluorescent biosensor market stood at approximately USD 2.7 billion in 2023 and is forecast to grow around 8% annually through 2028, potentially totaling USD 4.2 billion, driven by rising use in pharma, biotech, and healthcare. Advancements in nanotechnology, improvements in biosensor sensitivity and specificity, and broader adoption in point-of-care diagnostics are expected to significantly shape future market growth. Accordingly, the development of advanced fluorescent sensors remains one of the most promising strategies for protecting human health and the environment while meeting growing market demand.

Fluorescence relies on photon emission occurring within nanoseconds after an absorption event, giving these sensors a significant advantage in terms of rapid responsiveness. Moreover, fluorescence sensors can exploit the wavelength shift between absorbed and emitted light, enabling effective filtering of excitation light while accurately detecting the emitted fluorescence. As a result, fluorescent sensors provide highly accurate results with fast response times. The synthesis and development of advanced materials for fluorescence-based probes—capable of exhibiting distinct changes in fluorescence signals—have attracted considerable attention in the sensing field due to their high sensitivity, rapid response to analytes, and ease of operation [2,3]. In recent decades, a wide range of promising materials have been successfully developed and applied in the fabrication of fluorescent sensors, including conventional fluorophores [4], macrocyclic hosts [5], DNAzymes [6], Schiff-base ligands [7], metal–organic frameworks (MOFs) [8], small-molecule dyes [9], silica nanoparticles [10], core–shell materials [11], and copolymers [12]. Among the various potential materials, conjugated polymers (CPs) have emerged as promising luminescent candidates in the field of sensing, owing to their exceptional optical properties and high sensitivity [13]. These advantages stem from their strong fluorescence signal amplification capabilities, primarily attributed to the so-called “molecular wire effect.” This phenomenon enables efficient exciton migration along the polymer backbone, allowing even a single interaction event with an analyte to produce a pronounced fluorescence response. Such unique features make CPs superior fluorescent probes compared to small-molecule fluorophores, offering significant advantages for use in fluorescent sensor applications.

The development strategies of CPs for fluorescence sensors in environmental and biological applications mainly include three approaches: (i) selecting suitable monomers to tune absorption and emission properties; (ii) incorporating side chains to improve CP solubility; and (iii) functionalizing CPs with specific receptors to target particular analytes, thereby enhancing selectivity and sensitivity. A variety of CP-based fluorescent sensors have been developed using diverse polymer backbones and receptor moieties, and have been employed in biosensing and environmental analysis—including metal ion and anion detection, biomolecule sensing, bioimaging, and explosive detection—with high sensitivity and selectivity [14]. Many comprehensive reviews have been published on the applications of CP-based fluorescent sensors in the environmental and biological field [15,16,17,18,19,20,21,22]. Specifically, Hussain et al. conducted reviews on recent advances in CP-based sensors for monitoring various contaminants and metal ions [13]. However, there is a lack of focused reviews that specifically discuss the current challenges and bottlenecks, as well as provide a detailed assessment of the advantages and disadvantages of CP-based fluorescent sensors for environmental and biological applications. To consolidate current scientific advances in CP-based fluorescent sensors and to identify development trends, potential strategies, and emerging directions for future research, this study presents a comprehensive overview of CP-based fluorescent sensors. It emphasizes their unique properties and advantages, as well as recent innovations in the detection of environmental and biological analytes. Furthermore, it highlights the remarkable morphological and optical characteristics of CPs that enable the development of high-performance fluorescent sensors. The review also outlines the sensing mechanisms of CP-based fluorescent sensors for diverse environmental and biological targets and discusses the key factors that influence their sensing performance. Importantly, it provides a detailed comparison of recent research efforts, encompassing synthetic methods, material properties, and sensing characteristics of CP-based fluorescent sensors.

## 2. Fluorescent Properties of Conjugated Polymers

**Figure 1:**
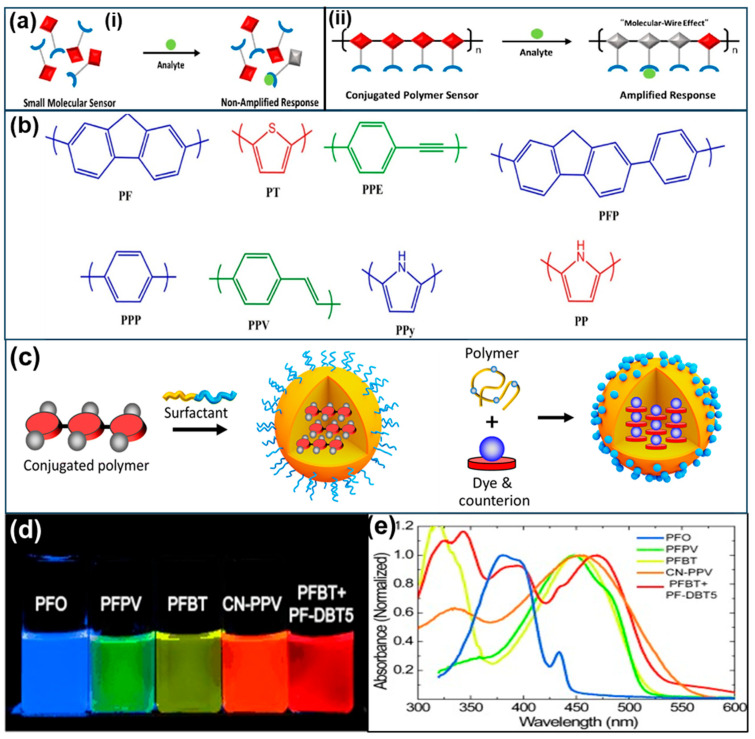
Schematic representation of (**a**) analyte-induced fluorescence quenching mechanism of CPs based on Swager’s molecular wire approach and: sensing amplification mechanism (i) without and (ii) with “molecular wire effect”(**b**) fundamental structures of some common CPs with variable backbones and their fluorescence color [13]; (**c**) the preparation process of fluorescent CPNs by loading cationic dyes [33]; (**d**) optical image of aqueous CP dot suspensions under UV light and (**e**) UV absorption spectra of CP dots in various solvents [32].

CPs possess semiconducting properties with high photo- and electroluminescence, as well as relatively high extinction coefficients across various wavelengths [23]. Their tunable band gaps can control the driving force of charge transfer reactions upon irradiation [24]. The optical properties of CPs can be modified through two main approaches: (i) chemical reactions and (ii) physical effects, such as changes in backbone planarity or strain [25]. Moreover, the color of CPs can be easily tuned by adjusting their conjugation length. Recently, CPs have garnered significant interest as emerging fluorescent materials due to their high fluorescence quantum yields and good photostability. Additionally, CPs can potentially amplify fluorescence signal responses through the “molecular wire effect” [26], further enhancing their utility in sensing applications (Figure 1a). Owing to these unique characteristics, certain CPs have been regarded as superior fluorescent probes for the design and fabrication of fluorescent sensors. Based on their polymer backbones, CPs can be categorized into several types with distinct color and fluorescence properties, including poly(p-phenylene) (PPP), poly(fluorene-co-phenylene) (PFP), polyfluorene (PF), polythiophene (PT), polypyrrole (PPy), poly(p-phenylene ethynylene) (PPE), and poly(p-phenylene vinylene) (PPV) (Figure 1b) [13]. For the synthesis of fluorescent CPs, transition-metal-catalyzed cross-coupling reactions have been commonly employed. These methods enable the efficient synthesis of diverse CP structures by incorporating various monomers [27,28]. These methods can simply control and modulate CP nanostructures and optical properties through selecting appropriate monomers and catalysts, as well as optimizing reaction conditions.

In general, the intriguing optical properties of CPs primarily arise from electronic delocalization and the mobility of charge carriers within their polymeric structures. As a result, CPs—often referred to as “amplifying fluorescent polymers”—can absorb radiation and return to their ground states via fluorescent emission. Consequently, CPs can be used in the design and fabrication of fluorescence sensors with significantly enhanced sensitivity due to the superquenching phenomenon. Meanwhile, the selectivity of CP-based fluorescence sensors can be improved by quencher–tether–ligand complexes which result from the conjugation between a quencher group in CP structures and a ligand of analytes [29]. Fluorescent CP sensors are often developed based on the deactivation or enhancement of their intrinsic fluorescence, referred to as “turn-off” or “turn-on” to sensing systems, respectively. CP sensors employing the fluorescence “turn-on” mechanism offer superior sensitivity compared to “turn-off” systems, primarily due to their low background fluorescence. Moreover, the former also reduces the likelihood of false-positive signals and enhances both selectivity and reliability, as fluorescence enhancement processes generally exhibit higher selectivity than fluorescence quenching processes. In general, the effectively amplified quenching effect in CPs is primarily attributed to the “molecular wire effect,” which arises from the high electronic mobility and efficient transport of excitons (electron–hole pairs generated through photoexcitation) along the polymer backbone. This electron-transfer quenching can lead to efficient deactivation of the excited state through trapping sites formed upon analyte binding. Moreover, the amplified quenching effect of CPs is strongly influenced by the length of their delocalized chains [30], increasing with the molecular weight of CPs. In terms of incorporating charged pendant groups, the CPs with water solubility showed high quenching efficiencies compared with neutral CPs. This phenomenon is ascribed to the formation of stronger complexes through electrostatic and hydrophobic interactions between the polyelectrolyte and quenchers. However, CPs with charged characteristics are self-assembly-aggregated in aqueous solutions, resulting in decreased fluorescence quantum yield by changing spectral properties [31]. The use of quencher ions can highly improve the quenching effects by promoting aggregation of CPs. The CP aggregation and quenched fluorescence is induced by interchain interactions and π-stacking of the CP backbones. The presence of several biological and chemical analytes can induce the analyte-induced aggregation processes of CPs [29].

Nanoparticles (NPs) derived from conjugated polymers (CPs), such as CP nanoparticles (CPNs) and conjugated polymer dots (CP dots, typically <30 nm in size), are emerging as promising nanomaterials with exceptional photophysical properties (Figure 1c). These materials offer several notable advantages, including tunable emission wavelengths, large molar extinction coefficients, high fluorescence quantum yields, and excellent photostability, making them highly versatile tools for a wide range of sensing applications [32,33]. In solution (e.g., tetrahydrofuran, THF), CP dots often exhibit broadened and blue-shifted absorption spectra (Figure 1d), which can be attributed to a decrease in conjugation length caused by bending, torsion, and kinking of the CP backbones [34,35]. The broad absorption bands of CP dots can range from λ = 350 nm to λ = 600 nm depending upon the specific polymers. Moreover, CP dots derived from different hydrophobic polymers exhibit multicolor emissions across the visible region (Figure 1e). In thin films, CP dots often exhibit a net redshift in their fluorescence spectrum due to energetic disorder and multiple energy transfer events.

Owing to the high extinction coefficient, high chromophore density, and high fluorescence quantum yield of CPs, CPNs are considered among the brightest fluorescent nanoparticles, being approximately 20–100 times brighter than similarly sized colloidal semiconductor quantum dots [36]. For the development and construction of highly sensitive and selective sensors, CPNs are often doped with organic dyes to induce a redshift in their fluorescence, thereby extending their emission range and enhancing detection capabilities [37]. Moreover, extensive efforts have been made to enhance the quantum yield and brightness of CPNs, enabling them to efficiently absorb and emit light in the visible region [38,39]. However, achieving high quantum yield emission in the far-red to near-infrared (NIR) region remains one of the major limitations in the development of CPNs, primarily due to aggregation-caused quenching effects associated with large π-conjugated polymer units. CPN-based probes emitting far-red to near-infrared (NIR) fluorescence are highly effective for bioimaging applications, owing to their low light scattering, deep tissue penetration, and favorable biocompatibility [40]. Therefore, bright CPNs with strong emissions in the far-red to NIR window have recently attracted significant attention for the development of CP-based fluorescent sensors. To fabricate these bright, long-wavelength emissive CPNs, donor–acceptor-type CPs with an optimized acceptor ratio have been widely adopted as a promising strategy and emerging direction. The size of CPNs is a critical factor that significantly influences their quantum yield and brightness [35], as larger particles tend to exhibit increased fluorescence quenching. Therefore, the preparation of bright red-to-NIR-emissive CPNs requires careful optimization of particle size, incorporation of donor–acceptor configurations, and the use of bulky side groups to reduce aggregation and enhance emission efficiency.

## 3. Working Mechanism of Conjugated Polymer Fluorescent Sensors

**Figure 2:**
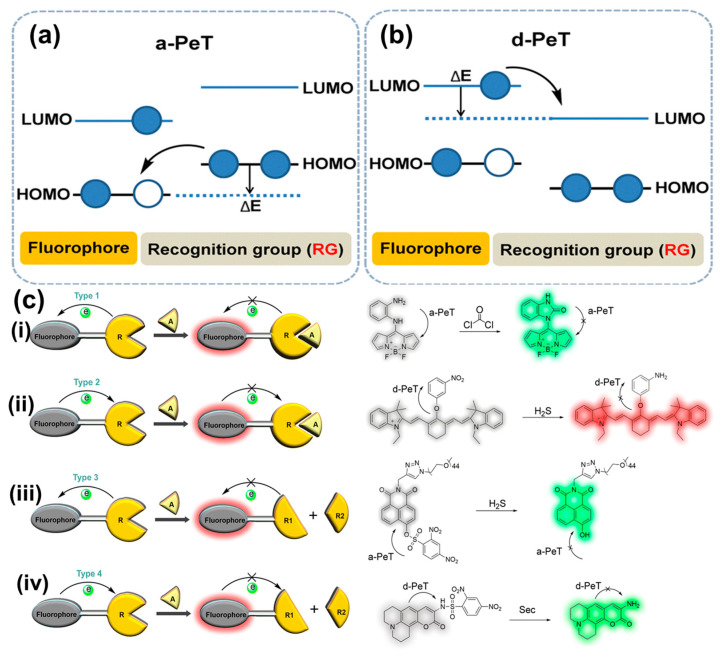
Schematic illustration of two main PET mechanisms, including (**a**) a-PET and (**b**) d-PET; (**c**) four design types of PET-based fluorescent CP probes: (i) Type 1, (ii) Type 2, (iii) Type 3, and (iv) Type 4 [50].

Similar to other fluorescent materials, CP-based fluorescent probes in sensors exhibit several primary sensing mechanisms, including photoinduced electron transfer (PET) [41], inner-filter effect (IFE) [42], Fȍrster resonance energy transfer (FRET) [43], and aggregation-caused quenching (ACQ) [44]. The CP-based fluorescent sensors typically respond immediately upon the addition of target analytes. The PET and FRET mechanisms rely on electron or energy transfer processes and, therefore, are highly dependent on the proximity between the CP probes and the analytes [45,46]. In contrast, the IFE mechanism is independent of this spatial relationship due to its characteristic reabsorption feature [47]. Meanwhile, the ACQ mechanism operates through fluorescence quenching of CP probes, caused by aggregation-induced π–π stacking of the CP backbones triggered by the presence of target analytes [48]. In general, a typical PET-based fluorescent probe normally comprises three basic elements: fluorophores, appropriate linkers, and recognition/activating groups [49]. During the PET process in CP-based fluorescent sensors, when analyte molecules encounter CP probes under photoexcitation, electrons are transferred from the donor (CP probe) at the highest occupied molecular orbital (HOMO) to the acceptor (analyte) at the lowest unoccupied molecular orbital (LUMO), resulting in quenching of the donor’s fluorescence [50]. Such electron transfer can only occur under the following conditions: (i) the energy levels of the donor and acceptor are well matched, and (ii) the donor and acceptor molecules are in close proximity, maintained by an existing cooperative interaction. To better understand the underlying principles governing this transfer phenomenon, the PET mechanism is often categorized into two subtypes based on the direction of electron transfer between the fluorophore and the recognition element: acceptor-excited PET (a-PET, Figure 2a) and donor-excited PET (d-PET, Figure 2b) [51]. In the a-PET mechanism, the HOMO level of the recognition group lies between the HOMO and LUMO levels of the fluorophore, enabling electron transfer from the recognition group (HOMO) to the partially vacant HOMO of the excited-state fluorophore. As a result, electrons in the fluorophore’s LUMO are unable to return to the ground state, leading to fluorescence quenching. Conversely, in the d-PET mechanism, the recognition group is electron-deficient, with its LUMO positioned between the HOMO and LUMO levels of the fluorophore. This allows electrons to transfer from the excited fluorophore to the recognition group. Specifically, electrons excited from the fluorophore’s HOMO to its LUMO undergo a non-radiative transition into the vacant LUMO of the recognition group, also resulting in fluorescence quenching. Furthermore, depending on the direction and nature of electron transfer between the fluorophore and the recognition group, PET-based fluorescent CP probes can be classified into four main types, based on changes in oxidation or reduction potential during the recognition process (Figure 2c). Type 1 represents the inhibition of the a-PET mechanism upon binding with analyte molecules, which is accompanied by an increase in the oxidation potential of the recognition group following recognition [52]. Type 2 involves the inhibition of the d-PET mechanism, accompanied by a decrease in the oxidation potential of the recognition group, rendering it incapable of efficiently accepting electrons [53]. In Type 3, the analytes cleave the recognition group, preventing the a-PET mechanism from occurring [54]. Finally, Type 4 involves the inhibition of the d-PET mechanism through analyte-induced cleavage of the recognition group [55]. In all four types, the fluorophore functions as either an electron donor or acceptor by being linked to electron-deficient or electron-rich moieties within the recognition group, leading to the activation or inhibition of d-PET or a-PET processes [56].

**Figure 3:**
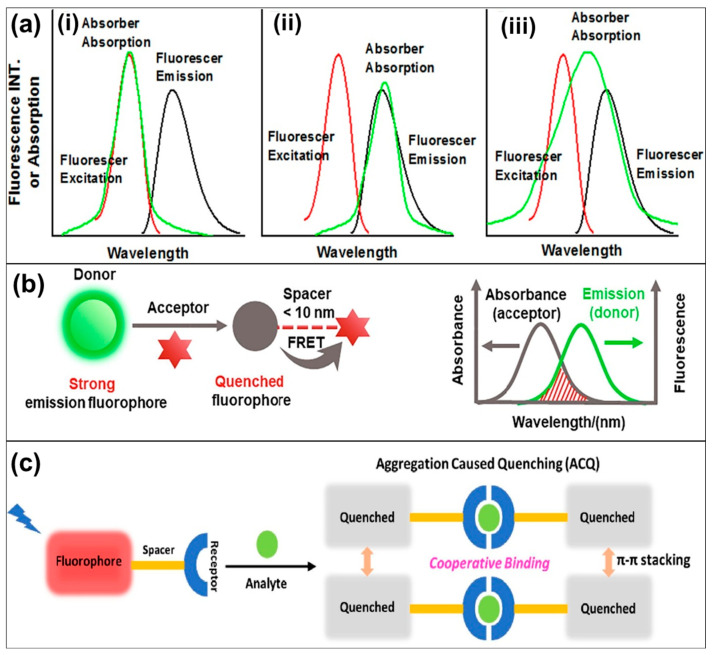
Schematic illustrations of several fluorescent mechanisms of CPs: (**a**) IFE mechanism and its operating spectral overlap conditions of the absorption spectrum of absorber with (i) absorption spectrum, (ii) emission spectrum, and (iii) both excitation and emission spectrum of fluorescer [47]; (**b**) FRET process [46]; and (**c**) ACQ process [13].

IFE was previously considered a source of error in fluorescence measurements; however, it has recently been recognized and effectively harnessed as a valuable non-radiative energy conversion mechanism for chemical and biosensing applications. Compared with traditional PET-based techniques, the IFE-based fluorescent approach offers greater flexibility and simplicity, as it does not require a direct linkage between the absorber and the fluorophore [57]. Based on whether the absorption affects the excitation or emission light, IFE mechanisms can be classified as primary or secondary IFE, respectively. Primary inner filter effect (pIFE) mechanism relies upon the absorption of excitation radiation, while secondary inner filter effect (sIFE) refers to the absorption of emission radiation. In general, an IFE-based sensing system, consisting of two optical components—an absorber and a fluorophore—can exhibit excellent sensing performance under the following conditions: (i) significant spectral overlap between the absorption spectrum of the absorber and the excitation and/or emission spectra of the fluorescer (Figure 3a); (ii) the absorber exhibits high sensitivity and selectivity toward various analytes through changes in its absorption spectrum, enabling quantitative detection of the target analyte; (iii) the fluorescence of the fluorescer remains independent of the analyte concentration, allowing it to function reliably as a fluorescent indicator in IFE-based sensors; (iv) the absorber possesses stable absorption and fluorescence properties that are minimally affected by external factors; (v) or it exhibits charge repulsion, preventing undesired interactions such as aggregation or energy transfer unrelated to the IFE process [47]. Therefore, the careful selection and combination of analyte-sensitive absorbers with analyte-independent fluorophores are critical for the effective design and performance of IFE-based sensors. The excitation/emission intensity of fluorescers can be modulated by varying the absorption characteristics of the absorber.

The FRET mechanism is one of the most commonly employed strategies in the design of CP-based fluorescent sensors. These sensors typically consist of a dyad of two fluorophores that form an energy donor–acceptor pair within a distance of approximately 1–10 nm [58]. In the FRET-based systems, the energy is transferred from the donor fluorophore to the acceptor fluorophore, which induces a shift in dual-emission intensities (Figure 3b). To ensure efficient operation of the FRET process, there must be sufficient spectral overlap between the emission spectrum of the donor and the absorption spectrum of the acceptor [59]. During the FRET process, interaction between the target analytes and the donor (probe) simultaneously leads to quenching of the donor’s fluorescence and enhancement of the acceptor’s fluorescence. These phenomena are attributed to non-radiative energy transfer from the excited-state donor (probe) to the ground-state acceptor (analyte). The non-radiative FRET process operates efficiently only under the following three conditions: (i) spectral overlap—there must be significant overlap between the emission spectrum of the donor and the absorption spectrum of the acceptor; (ii) proximity—the donor and acceptor must be within a distance of approximately 1–10 nm; and (iii) donor quantum yield—the donor molecules must possess a sufficiently high quantum yield to facilitate effective energy transfer. Besides the main FRET mechanism, two other energy transfer processes—Dexter energy transfer (DET) and through-bond energy transfer (TBET)—have recently been proposed as potential mechanisms underlying the sensing behaviors of CP fluorescent sensors. DET is generally defined as an electron-exchange process that involves p-orbital overlap [60]. The efficiency of DET in optical devices, especially fluorescent sensors, is favored by short intermolecular spacing and the presence of a triplet exciton transition between the sensitizer and the final emitter. However, this process can accelerate the degradation of the emitter material and thereby influence the operational lifetime of fluorescent sensors [61,62]. Consequently, the development of highly efficient and durable fluorescent sensing devices requires minimizing DET while simultaneously enhancing FRET [63]. Meanwhile, TBET is a nonradiative energy transfer mechanism in which donor excitation energy is delivered to a nearby acceptor through a rigid, electronically conjugated π-system linker that disrupts the coplanarity of the donor and acceptor groups [64]. Fluorescent sensors operating via the TBET mechanism are mainly composed of three parts: an energy donor, an energy acceptor, and a rigid linker. In contrast to the commonly used FRET mechanism, TBET does not require spectral overlap, which allows for a broader range of donor–acceptor combinations and provides higher sensitivity toward targets through ratiometric fluorescence [65].

Finally, the ACQ mechanism—well known as a common fluorescence quenching phenomenon—primarily arises from the non-radiative decay of excited-state fluorophores when they form aggregates [66]. In other words, the fluorescence quenching effect of ACQ mainly relies on the aggregation-induced π-π stacking of the CP molecules often triggered by the presence of target analytes (Figure 3c) [48,67,68]. H-aggregation in the structure of CPs is often considered a key factor responsible for inducing ACQ. Analysis of intermolecular interactions and reorganization energy has shown that the enhanced contribution of C–H stretching vibration modes is primarily driven by intermolecular hydrogen bonding formed in the aggregated state. Therefore, fluorescent efficiency can be effectively improved by preventing the formation of hydrogen bonds during aggregation [69]. In addition, increasing the hydrophobicity and molecular planarity of CPs promotes π–π stacking, thereby improving water sensitivity and strengthening the cohesion of quenched aggregates. Thus, the development of novel CP fluorescent probes with absolute ACQ (aACQ)—achieved by rigidifying the aza-BODIPY parent structure and/or introducing methoxy or acetoxy groups—has been proposed as a promising strategy to minimize fluorescence retention and re-illumination while simultaneously enhancing ACQ efficiency [70].

## 4. Advantages and Disadvantages of Conjugated Polymers in Fluorescent Sensors

Thanks to unique optical and electrochemical properties, CPs inherently exhibit fluorescence-based responses, including “turn-on” and “turn-off” behaviors during target recognition [20]. In particular, CP-based fluorescent probes and devices have been widely developed across various sensory systems and transduction mechanisms. Compared to other common fluorescent materials such as organic dyes, quantum dots, and metal nanoclusters, CPs exhibit attractive features, including sensory signal amplification, tunable structures, adjustable absorption/emission wavelengths, and controllable energy offsets, making them particularly suitable for diverse sensing applications. This versatility has made CPs a preferred fluorescent sensing platform for environmental and biological applications. However, CPs also face notable drawbacks, particularly the challenges in designing suitable frameworks and optimizing their fluorescence properties. Many CPs are hydrophobic and exhibit low solubility in aqueous solutions, which restricts their direct application in biological systems. In addition, compared with small organic dyes or inorganic quantum dots, their operational lifetime can be shorter without proper stabilization strategies, as they are prone to photobleaching under prolonged excitation and degradation under environmental conditions such as oxygen, moisture, or pH fluctuations. Table 1 highlights and compares several important properties, advantages, and disadvantages of CPs and other materials in the design and construction of fluorescent sensors.

## 5. Recent Advances and Innovations in CP-Based Fluorescent Sensors in Environmental and Biological Applications

### 5.1. Conjugated Polymer-Based Fluorescent Probes for Detection of Metal Ions

Enormous amounts of metal pollutants are discharged annually, imposing a heavy burden on the environment and causing harmful effects on human health [71,72,73]. In particular, heavy metal ions can accumulate in living organisms through the direct intake of contaminated water or via the food chain. Consequently, they can disrupt the metabolism of living organisms, damage cellular organelles, and impair enzymes involved in metabolic processes, signaling pathways, and cellular repair mechanisms [74]. Moreover, they are highly stable and resistant to degradation, making them toxic to the human body [75,76]. Even exposure to low levels over prolonged periods can lead to numerous adverse health effects [77]. Therefore, there has been increasing attention and growing research interest in the design and fabrication of biological and chemical materials for the detection of low concentrations of metal ions [71]. Among various sensing techniques, fluorescent probes based on CPs have emerged as one of the most attractive methods for detecting metal ions due to their superior advantages, including high sensitivity, excellent selectivity, portability, and cost-effectiveness. Common fluorescent CPs such as polyacetylene, polyfluorene, polythiophene, and polyphenylene have been widely used as optical sensing materials for the detection of metal ions, owing to their unique optical properties, self-assembly capabilities, and tunable structures and properties. Various backbone structures of CPs have been developed and utilized in designing highly sensitive probes for the detection of numerous metal ions, exhibiting different inherent fluorescent behaviors, including “turn-on” and “turn-off” responses during target recognition [20]. For instance, common CPs such as polydiacetylene and polythiophene, which possess specific backbone structures, exhibit significant shifts in absorption and emission wavelengths upon interaction with target analytes [78]. In general, CPs have been functionalized at their side chains with various groups—such as ionic moieties, quenching groups, organic dyes, and responsive units—to further enhance their sensing capabilities [68,79]. Owing to signal amplification and the molecular wire effect—achieved through backbone design and side-chain functionalization—CP-based sensors often exhibit ultrahigh sensitivity to metal ions, with detection limits ranging from the micromolar to nanomolar, and even picomolar, levels. Moreover, by tailoring the water solubility of the main chain, several hydrophilic CP-based probes have been developed for the detection of metal ions in aqueous solutions, offering promising potential for applications in diagnostics and bioimaging. Finally, bicolor fluorescent sensors based on CPs have recently demonstrated high efficiency in identifying different metal ions [80,81].

Recent progress and applications of CP-based fluorescent probes for metal ion sensing are summarized in Table 2.

**Figure 4:**
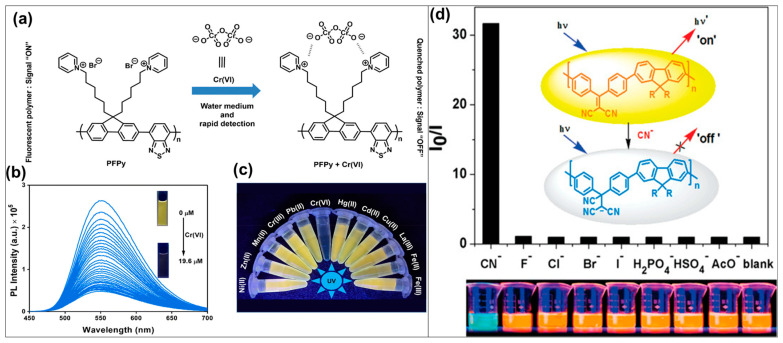
Schematic illustration of (**a**) chemical structure of PFPy and its fluorescent sensing mechanism; (**b**) fluorescence spectra of PFPy and (**c**) photograph of PFPy solution over various concentration of dichromate [129]; (**d**) fluorescence responses and emission images of the conjugated polyfluorene polymer sensor upon exposure of different anions (h*v* and h*v*’represent excitation photon and emission photo) [130].

Many effective CP fluorescent sensors have been successfully developed for the recognition of cationic or neutral metal species. However, their application to the detection of metal anions remains limited, even though many anions play critical roles or exhibit high toxicity in numerous biological and chemical processes. Recently, several CPs have been developed and effectively employed for detecting various important metal anions such as dichromate, fluoride, and cyanide. In these CP fluorescent sensors, metal anions interact with CPs through covalent or noncovalent (displacement approach) bindings to induce the changes and signals [126]. Dichromate (Cr_2_O_7_^2−^) is a widespread contaminant in wastewater and can remain stable in water for extended periods, thereby posing a serious threat to the health of humans and other organisms [127]. The development of efficient sensors based on fluorescent CPs for Cr_2_O_7_^2−^ is highly desirable [128]. For example, Tanwar et al. reported a highly sensitive and selective poly(1,1′-((2-(benzo[c][1,2,5]thiadiazol-4-yl)-9H-fluorene-9,9-diyl)bis(hexane-6,1-diyl))bis(pyridin-1-ium)dibromide) (PFPy) sensor for the rapid detection of dichromate anions in water, as well as on cost-effective portable paper test strips (Figure 4a–c) [129]. In this sensor, PFPy exhibited an excellent fluorescence “turn-off” signal upon exposing dichromate anions, operating through a dynamic fluorescence quenching mechanism, with an LOD of ~6.79 ppb—significantly lower than the safety level recommended by the WHO. Moreover, the PFPy sensor was successfully applied to the detection of dichromate anions in real environmental water samples and demonstrated good cost-effectiveness for monitoring dichromate in drinking water in rural areas. Cyanide (CN^−^), a highly toxic anion capable of causing human fatality within minutes, has recently attracted considerable attention and emerged as a potential target contaminant in numerous studies involving CP fluorescent sensors. For instance, Wu and coauthors developed a CP chemosensor for highly selective and sensitive detection of cyanide (Figure 4d) [130]. This fluorescent sensor was constructed from a conjugated polyfluorene containing the dicyanovinyl group which can act as a selective cyanide-reactive unit through the nucleophilic addition reaction. This reaction could induce changes in CP main chains and their electronic structure and optical properties. Moreover, due to the excellent exciton-transporting properties of the synthesized CP and the charged nucleophilic reaction, the fluorescence quenching effect can be significantly amplified and the fluorescent sensor exhibited higher sensitivity and selectivity toward CN^−^. Besides dichromate and cyanide, the fluoride anion (F^−^) has also emerged as an attractive target for CP fluorescent sensors due to its significant implications in health and environmental issues, including osteoporosis treatment, dental care, and water supply management. Qu et al. reported a diketopyrrolopyrrole (DPP)-based fluorescent CP sensor capable of amplifying the detection of fluoride ions and demonstrating potential applications in bioimaging [131]. The authors employed copolymerization of DPP and fluorene monomers in the main chain to construct a delocalized electronic structure within the CP, thereby providing emission traps that facilitate exciton transport in the excited state. The fluoride anion acts as a quencher through strong deprotonation interactions, which disrupt the electronic energy transfer process. This resulted in a pronounced fluorescence quenching accompanied by a distinct red-to-purple color change in the CP solution, demonstrating its great potential as a sensor for fluoride ion detection.

### 5.2. Conjugated Polymer-Based Fluorescent Sensors for Detection of Explosives

Explosives belong to the broad family of neutral chemical species and are primarily classified into four main categories: (i) nitroaromatics (e.g., 2,4,6-trinitrotoluene [TNT], 2,4-dinitrotoluene [2,4-DNT], and picric acid [PA]) and nitroalkanes (e.g., 2,3-dimethyl-2,3-dinitrobutane [DMNB]); (ii) nitramines (e.g., 1,3,5- trinitro-1,3,5-triazacyclohexane [RDX]); (iii) nitrate esters (e.g., pentaerythritol tetranitrate [PETN]); and (iv) peroxides [132]. The strong oxidizing nature of the explosives has been often exploited to design fluorescent probes based on redox reactions, accompanied by color or emission changes. Moreover, many explosives act as efficient quenchers of fluorescence through photoinduced electron transfer (PET) processes, due to their pronounced electron-deficient character imparted by nitro-functionalized organic structures. Therefore, there has been growing interest in the development of fluorescent amplifying CPs for explosive detection. CPs are well-suited materials for the fabrication and development of optical sensors for explosives, particularly for the detection of nitroaromatic compounds [133]. Upon light absorption, the polymer generates an exciton that emits fluorescence; however, electron transfer from the polymer to a sorbed nitroaromatic compound can dissociate the exciton, resulting in a measurable decrease in fluorescence intensity [134]. CPs often exhibit good sensitivity to explosives in the vapor phase, but practical detection in the aqueous phase remains challenging, primarily due to limitations in their photophysical and mechanical stability.

Recently, microporous fluorescent CPs have been employed as sensory materials for nitroaromatic explosives, owing to the synergistic combination of their porous morphology and rapid exciton migration facilitated by long-range intermolecular π–π stacking [135,136]. CPs have been widely utilized in the development of highly sensitive and selective biosensors for detecting various explosives, as summarized in Table 3. Conjugated microporous polymers (CMPs), a type of amorphous porous organic polymer, have emerged as promising fluorescent sensors due to their exceptional hydrothermal stability and structural versatility. Moreover, CMPs show strong potential as reversible fluorescent sensors, attributed to their permanent porosity, π-conjugated frameworks, tunable backbones, and inherent capacity for host–guest interactions [137]. CMPs feature π-conjugated linkages and three-dimensional networks, and their fluorescent variants have attracted significant attention for the accurate detection of explosives, owing to their high surface area, permanent porosity, and excellent chemical and physical stability [138]. Alongside CMPs, conjugated polymer nanoparticles (CPNs) have recently emerged as a promising class of nanomaterials for the highly sensitive detection of explosives, owing to their high quantum efficiency, low cytotoxicity, exceptional sensitivity, and outstanding photostability against photobleaching [15,139]. CPNs have been demonstrated as a highly efficient and reliable platform for detecting trace amounts of the nitroexplosive picric acid across multiple media and forms—including solutions (various solvents), disposable paper films (solid/powder), and devices (vapor/gas)—offering ultrasensitivity and strong, unambiguous signal responses [140]. CPNs, primarily prepared by emulsion or reprecipitation of linear CPs [32], have shown limited success as fluorescent sensors for nitroaromatic explosives, with challenges remaining in both sensitivity and selectivity [141,142].

**Figure 5:**
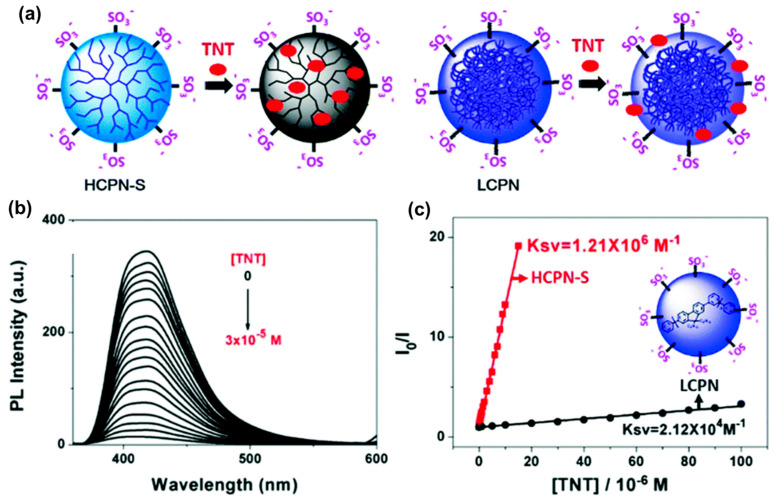
(**a**) Schematic illustration of the proposed structures and sensing mechanisms of HCPNs and LCPs for TNT detection; (**b**) fluorescence spectra of HCPN dispersions at an excitation wavelength of 340 nm in response to varying TNT concentrations; (**c**) Stern–Volmer plots showing fluorescence quenching of HCPNs at 419 nm and LCPs at 394 nm by TNT [143].

To address the limitations and challenges associated with CPNs derived from linear conjugated polymers (LCPs), hyperbranched conjugated polymer nanoparticles (HCPNs) have recently been developed and introduced as fluorescent sensors [143]. Although numerous fluorescent sensing materials have been reported for the detection of nitroaromatic explosives, most are based on LCPs. In contrast, HCPNs are highly branched organic semiconductors with dendritic, multidimensional architectures and a high density of reactive terminal groups. Their delocalized electronic structure facilitates efficient coupling among sensing-relevant structural units, thereby enhancing their performance as fluorescence-based sensors. The synthesis of HCPNs allows control over the molar mass and branching degree of conjugated polymers (CPs), distinguishing them from both their linear analogs and perfectly branched dendrimer counterparts. Moreover, HCPNs can be synthesized in a controlled and scalable manner, making them promising candidates for fluorescence-based sensors targeting explosive-induced quenching. From a theoretical perspective, HCPNs are expected to exhibit greater sensitivity than LCPs. In linear polymers, when a specific site interacts with a quenching agent, exciton quenching occurs within the exciton diffusion length (EDL) [20]. In contrast, HCPNs contain multiple conjugated chains within the EDL range from the point of contact, enabling more efficient quenching of the exciton generated at that site. Consequently, it is hypothesized that the quenching rate in HCPNs is significantly higher than in LCPs [144]. Based on these hypotheses, Wu and coauthors developed a novel water-dispersible HCPN with highly amplified fluorescence sensing capability by combining hydrophobic CP cores with hydrophilic sulfonate terminal groups, enabling highly sensitive detection of TNT (Figure 5a) [143]. Due to the efficient encapsulation of TNT within numerous hydrophobic cavities of the hyperbranched CP core, this HCPN exhibited a quenching constant of 1.21 × 10^6^ M^−1^—significantly surpassing those of conventional CP analogs by two to three orders of magnitude (Figure 5b,c). This enhanced quenching efficiency enabled highly sensitive detection of TNT, with a detection limit as low as 3.7 nM. Additionally, HCPN-S exhibited strong selectivity for TNT, maintaining its sensing performance even in the presence of 2,4,6-trinitrophenol and various other nitroaromatic compounds.

**Table 3:** Summary of recent applications of CP-based fluorescent biosensors for the detection of various explosives.

CPs	Types of Sensors	Explosives	LOD	Refs
Poly [2-methoxy-5-(2-ethylhexyloxy)-1,4-phenylenevinylene]	Fluorescence quenching sensor	1,4-dinitrobenzene	6.3 ppm	[145]
PDY-132 (Super Yellow) *	Fluorescence sensor	2,4-DNT	8.17 μM	[134]
TBP–BB *	Fluorescence probe	2,4,6-trinitrophenol	0.1 mM	[137]
Poly(9,9-dioctylfluorene2,7-diyl)-co-bisthiophene	Handheld explosive sensor	Nitrobenzene	17 μM	[146]
Oligo(p-phenylenevinylene) gelator	Fluorescence sensor	Trinitrotoluene (TNT) Dinitrotoluene (DNT)	5 ppb	[136]
Fuorene-based CPs	Colorimetric and fluorometric sensor	2,4,6-trinitrophenol (TNP)	3.2 pM	[147]
Poly(3,3′-((2-phenyl-9H-fluorene-9,9-diyl)bis(hexane-6,1-diyl))bis(1-methyl-1H-imidazol-3-ium)bromide)	Fluorescence sensor	Nitroexplosive picric acid	30.9 pM	[140]
Poly(aryleneethynylenesiloles)	Fluorescent chemosensors	Picric acid TNT	10 μM	[148]
Folded tetraphenylethene	Fluorescent chemosensors	Picric acid	0.57 mM	[149]
Conjugated microporous polymers	Fluorescent chemosensors	TNT	5.85 μM	[138]
9,9-dioctyl-2,7-dibromofluorene	Fluorescent chemosensors	TNT	1.6 μM	[150]
Polycarbazole	Fluorescent chemosensors	TNT DNT	5 ppb 100 ppb	[151]
Poly(dichloromethylphenylsilane)	Fluorescent chemosensors	TNT	1.76 μM	[152]
Poly(2,3,5,6-styrylpyrazine)	Fluorescent quenching sensor	TNT	1.76 μM	[144]
Conjugated porous polymer (CPP)	Fluorescent quenching sensor	TNT	5 ppb	[153]
Hyperbranched conjugated polymer nanoparticle	Fluorescent chemosensors	TNT	3.7 nM	[143]
Poly(1,4-divinyl-2,5-dioctyloxybenzene-alt-5,8-dibromo-2,3-diphenylquinoxaline)	Fluorescent quenching sensor	TNT	5 ppm	[154]
Donor–acceptor polymer	Fluorescent quenching sensor	Picric acid TNT	2 ppb 23 ppb	[155]
2,2’-biimidazole-containing conjugated polymer	Fluorescent quenching sensor	Picric acid	0.51 μM	[156]
Di(naphthalen-2-yl)-1,2-diphenylethene	Fluorescent chemosensors	Picric acid	0.181 μM	[157]
Poly [4,4′-(((2-phenyl-9H-fluorene-9,9-diyl)bis(hexane-6,1-diyl))bis(oxy))dianiline)]	Fluorescent chemosensors	Picric acid	57.8 nM	[158]
PTPA-TPE	Fluorescent quenching sensor	Picric acid	72 ppb	[159]
Polybenzimidazole	Fluorescent quenching sensor	DNT	3.3 ppb	[160]
Polycarbazole	Fluorescent quenching sensor	DNT	-	[161]
2,7-dibromo-9,9-dihexylfluorene	Fluorescent quenching sensor	DNT	0.4 mM	[162]
1,1,2,2-tetrakis(4-formyl-(1,1′-biphenyl))-ethane	Fluorescent quenching sensor	TNP	0.09 μM	[163]
Poly(triphenylamine-co-benzothiadiazole)s	Fluorescent chemosensors	1,3,5-trinitrobenzene (TNB)	19 μM	[164]
Polycarbazoles	Fluorescent quenching sensor	TNB	50 nM	[165]

* PDY: Poly [2,5-di(3,7-decyloxy)-1,4-phenylenevinylene-co-3-(4′-(3′,7″-decyloxy)phenyl)−1,4-phenylenevinylene-co-3-(3′-(3′,7-decyloxy)phenyl)-1,4-phenylenevinylene]; TBP–BB: 3,10,15--3,10,16-tribromotrinaphtho [3.3.3]propellane (TBP) and 4,7-bis(4,4,5,5-tetramethyl-1,3,2-dioxaborolan-2-yl)-2,1,3-benzothiadiazole (BB).

### 5.3. Conjugated Polymer-Based Fluorescent Sensors for Detection of Pathogens

**Figure 6:**
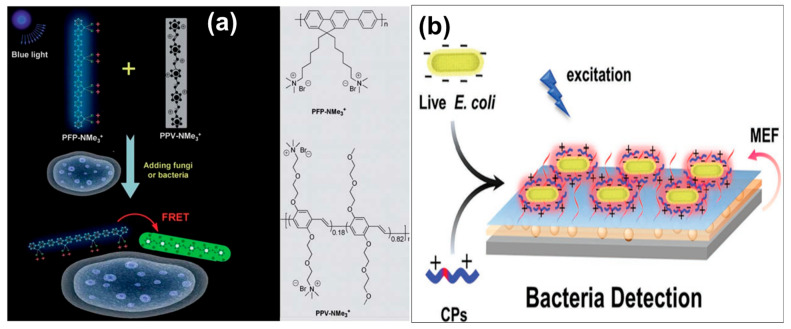
Schematic representations of (**a**) pathogen detection based on Förster resonance energy transfer (FRET) of cationic conjugated polymers, i.e., PFP-NMe_3_^+^ and PPV-NMe_3_^+^ [170], and (**b**) the bioassay structure and mechanism for *E. coli* detection, showing enhanced fluorescence intensity of CPs due to the metal-enhanced fluorescence (MEF) effect of Ag nanoparticles (Ag NPs) [171].

Pathogenic microorganisms can enter the human body via air, contaminated water, food, or medical instruments, leading to infections and infectious diseases [166]. Bacteria, fungi, and viruses are common pathogenic microorganisms that pose serious threats to human health and life through major outbreaks of infectious diseases—such as tuberculosis, cholera, severe acute respiratory syndrome (SARS), influenza A, and Ebola—which have rapidly become global threats, particularly in the context of increasing globalization. Most recently, severe acute respiratory syndrome coronavirus 2 (SARS-CoV-2) has posed a significant threat to global health through the widespread transmission of the pandemic known as coronavirus disease 2019 (COVID-19) [167]. Therefore, rapid and accurate detection of pathogens, along with effective discrimination among pathogenic microorganisms, has become a top priority in the fight against their associated infections. To meet the high standards required for such rapid detection and identification, numerous materials and methods have been developed and optimized. Among these materials, CPs have attracted widespread attention in this field due to their exceptional light-harvesting properties and strong optical signal amplification capabilities [168]. CPs have recently been utilized in the development of highly sensitive and selective biosensors for the identification of various pathogen strains, as summarized in Table 4. Even single CPs without specific recognition groups can effectively enable direct and rapid detection and discrimination of pathogens, owing to structural variations on pathogen surfaces and differences in their interactions with CPs [169]. For instance, Wang and coworkers prepared two cationic donor–acceptor CPs, i.e., PFP–NMe_3_^+^ and PPV–NMe_3_^+^, that operate via a FRET mechanism to enable highly effective detection and discrimination of bacteria and fungi (Figure 6a) [170]. To prevent nonspecific adsorption onto pathogen surfaces, the side chain of PPV–NMe_3_^+^ was covalently modified with polyethylene glycol (PEG) groups, allowing the two cationic CPs to interact differently with negatively charged species. Based on zeta potential measurements, PPV–NMe_3_^+^ demonstrated higher binding selectivity for *Escherichia coli* (*E. coli*) than for *Candida albicans* (*C. albicans*), whereas PFP–NMe_3_^+^ showed greater selectivity for the latter over the former. The unequal distribution of the donor (PFP–NMe_3_^+^) and acceptor (PPV–NMe_3_^+^) on the surfaces of *E. coli* and *C. albicans* was considered the main reason for the observed differences in FRET efficiencies. In another study, Wang et al. successfully developed a multifunctional bioassay platform based on CP–silver nanostructure pair for detection of bacteria [171]. The bacteria (*E. coli*) can be captured on the platform surface via interactions with the conjugated backbone and hydrophilic side chains of the cationic CPs, which induces a fluorescence response (Figure 6b). When coupled with plasmon-enhanced fluorescence from the Ag nanostructure, this process forms a bioassay platform for bacterial detection. This phenomenon, referred to as metal-enhanced fluorescence (MEF), is strongly influenced by the distance between the fluorophore and the metal nanoparticle. Fluorescence enhancement occurs only when this distance falls within an optimal range relative to the metallic nanostructure.

### 5.4. Conjugated Polymer-Based Fluorescent Sensors for Detection of Other Biomolecules

**Figure 7:**
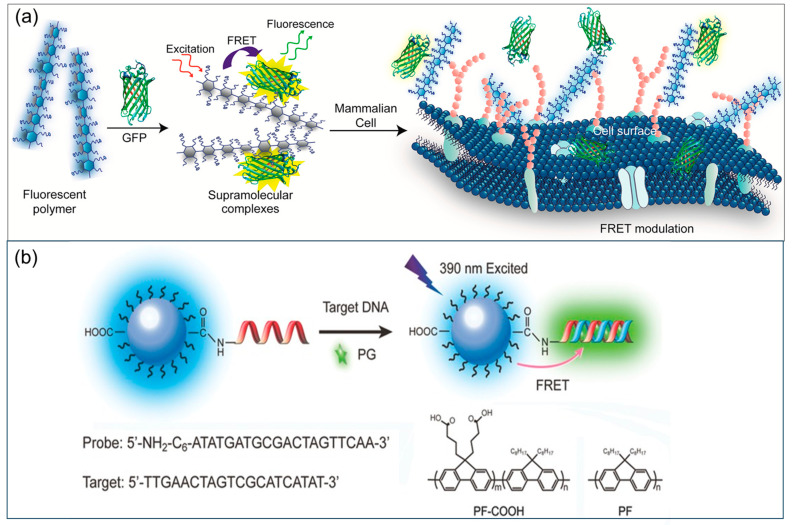
Schematic illustration of (**a**) a FRET sensor based on complexes of CP and green fluorescent protein (GFP) for cell sensing [185]; (**b**) CP nanoparticle-based fluorescent sensor for label-free DNA detection [188].

Recently, CPs have been employed to synthesize water-dispersible fluorescent nanoparticles for both in vitro and in vivo sensing and bioimaging applications, highlighting their great potential as fluorescent probes due to their high brightness, excellent photostability, and low cytotoxicity [183]. These CP nanoparticles with bright FR/NIR fluorescent characteristics often exhibit high quantum yield (~25%) in water because of taking advantage of the efficient intramolecular and intermolecular energy transfer. Furthermore, they have demonstrated promising applications as in vivo probes for the effective diagnosis of various disease biomarkers, including those relevant to clinical cancer diagnostics. For instance, Liu et al. synthesized an FR/NIR fluorescent CP, designated PFDBT-POSS, consisting of a poly [9,9-di(hexyl)fluorene]-alt-co-[4,7-bis(thiophen-2-yl)-2,1,3-benzothiazole] (PFDBT) backbone with bulky polyhedral oligomeric silsesquioxane (POSS) side chains, and subsequently employed it to construct a fluorescent sensor for the accurate diagnosis and imaging of targeted cancer cells through click reaction with an anti-HER2 affibody [184]. The results indicated that the PFDBT-POSS–affibody conjugate could reliably detect and image HER2-overexpressing cancer cells, serving as a fluorescent probe with low cytotoxicity and excellent photostability. In another study, Rana and coauthors constructed a FRET-based ratiometric biosensor array using a family of positively charged CPs and green fluorescent protein (GFP), and then used it to detect different mammalian cell surfaces (Figure 7a) [185]. This study established a universal platform for the rapid (within minutes) and highly sensitive (down to ~2000 cells) identification of various mammalian cell types by exploiting their unique “fingerprint” surface phenotypes, without the need for specific biomarkers or external cell labeling. Moreover, the sensor array demonstrated a strong capability to detect overall molecular differences on cell surfaces and to differentiate between isogenic cells with distinct glycosylation patterns, thereby offering new opportunities for cancer diagnostics based on glycan biomarkers.

More recently, to meet the demand for facile and specific DNA sensing, CPs have been surface-functionalized and assembled into CP nanoparticles, thereby enhancing their practicality in label-free DNA-related assays. Nowadays, some common methods have been employed to functionalize the CP surface and nanoparticles, including phospholipid encapsulation [186], co-condensation of amphiphilic polymers [183], and surfactant mini-emulsion [187]. Bao et al. reported a simple bioconjugate-recognized DNA sensor based on polyfluorene CP nanoparticles functionalized with anionic carboxylic acid groups on their surface (Figure 7b) [188]. In this study, the hybrid bio/synthetic polyfluorene nanoparticles enabled selective, label-free detection of target ssDNA in serum. Moreover, functionalization with carboxylic acid groups allowed the anionic polyfluorene nanoparticles to serve as signal-amplifying units through bioconjugation with amine-functionalized single-stranded oligonucleotides acting as receptors. In addition, the presence of target ssDNA induced a distinguishable fluorescent color change in the DNA-bioconjugated CP nanoparticles, enabling the possibility of naked-eye detection of ssDNA under UV irradiation. Compared with conventional conjugated polyelectrolytes that rely on electrostatic attraction for DNA detection, this CP sensor design demonstrated superior sensitivity for anionic DNA, attributable to enhanced interchain interactions and more efficient exciton transport within the nanoparticles. Moreover, owing to the covalent DNA coating, the photostability of CP nanoparticles under different buffer conditions was significantly enhanced, which is highly desirable for diverse applications and enables their use as sensors in physiologically relevant environments.

Meanwhile, water-soluble cationic CPs—such as poly(fluorene phenylene), polyfluorene, and polythiophene—have recently been employed as fluorescent probes for the fabrication of DNA- and aptamer-based biosensors, owing to their excellent light absorption and emission properties [189], as well as charge attraction [168]. For example, Gaylord et al. introduced a FRET-based DNA sensor using cationic CPs to sensitize the emission of fluorescein-tagged peptide nucleic acids [190]. This cationic CP sensor exhibited very high sensitivity with LOD of 10 pM target DNA and 25-fold higher fluorescence emission, compared with other sensors obtained by fluorescein only. Meanwhile, He and coauthors used ethidium bromide (EB), known as a duplex intercalator, to prepare a FRET sensor for detecting the transition of DNA in real time through interaction between cationic CPs and fluorescein, and EB [191]. In another approach, Wang and coworkers employed cationic CPs to construct a high-performance fluorescent sensor for detecting genetic disorders, including DNA methylation and single-nucleotide polymorphisms [192]. In this strategy, single-base extension or polymerase chain reaction (PCR) was incorporated to generate large amounts of fluorescein-labeled double-stranded DNA (dsDNA), enabling detection through the FRET signal arising from the complex formed between CCPs and dsDNA. However, these FRET-based sensors required the use of DNA-intercalating dyes or covalent DNA labeling. Very recently, the cationic CP poly(3-(3′-N,N,N-triethylamino-1′-propyloxy)-4-methyl-2,5-thiophene) (PMNT) was employed to design a label-free fluorescent biosensor [193]. This work elucidated the interactions between PMNT and DNA during the sensing process, in which the fluorescence of PMNT at 530 nm initially decreased, followed by the emergence of a new peak at 580 nm upon binding with ssDNA. Moreover, the binding force between PMNT and DNA was initially dominated by electrostatic interactions, while DNA base-mediated interactions became increasingly important at later stages. Owing to the crucial role of electrostatic interactions in the sensing mechanism, the performance of the PMNT fluorescent sensor was highly dependent on salt concentration and pH, with optimal conditions at 0.3 M NaCl and pH 6–8. In addition, both the sequence and length of DNA influenced the sensitivity. Under these optimized conditions, the PMNT-based biosensor achieved an excellent LOD of 1 nM for DNA.

## 6. Conclusions and Perspectives

CPs, with their excellent optical and fluorescent properties as well as good flexibility, are among the most suitable materials for fabricating high-performance fluorescent sensors for environmental and biological applications. CPs have been increasingly utilized in the development of highly sensitive and selective sensors targeting a wide range of compounds, including heavy metal ions, explosives, and pathogenic microorganisms, offering high accuracy and rapid response. Compared to other fluorescent materials—such as metal nanoclusters, organic dyes, and quantum dots—CPs offer several attractive features, including sensory signal amplification, tunable molecular structures, adjustable absorption and emission wavelengths, and controllable energy offsets. These properties make CPs particularly well suited for a wide range of sensing applications. This versatility has established CPs as a preferred platform for fluorescent sensing in both environmental and biological contexts. Moreover, CPs have been continuously evolving into various unique nanostructures and hybrid forms to meet the stringent standards and requirements of next-generation portable sensing technologies. The detection capability of CP-based fluorescent sensors can be realized in both liquid and solid states, in aqueous or organic environments, and even within complex matrices such as blood serum. Leveraging the unique properties of CPs, a wide range of analytes—including nitroaromatic explosives, heavy metal ions, pathogens, cyclic ketones, plant hormones, short peptides, DNA strands, and enzymes—have been successfully detected with high sensitivity and selectivity. Moreover, in some cases, CP-based fluorescent sensors have enabled naked-eye detection of target analytes, even under extremely dilute conditions.

With the continuous advancement of smart and innovative sensing technologies, functional materials are being increasingly developed and applied in flexible and wearable devices. From a materials perspective, CPs can be integrated with other promising materials—such as MXenes, perovskites, quantum dots, graphene, carbon dots, and other carbon-based nanomaterials—to enhance their optical properties and functionalities, thereby offering improved sensitivity and selectivity toward target analytes. Moreover, these integrations can help meet the stringent requirements of next-generation portable and wearable sensors for environmental and biological applications. The incorporation of these advanced materials is also expected to drive significant miniaturization of CP-based fluorescent sensing devices, resulting in enhanced wearability, streamlined fabrication processes, and improved suitability for long-term use due to reduced skin irritation.

To address the current disadvantages and challenges in the development of CPs for fluorescent sensing applications, several promising strategies and perspectives have been proposed. First, water solubility and biocompatibility can be improved through the following approaches: (i) introducing ionic, zwitterionic, or hydrophilic side chains (e.g., sulfonate, PEGylation) to enhance dispersibility in aqueous solutions; (ii) developing novel nanoparticles, micelles, or CP dots using amphiphilic copolymers for stable dispersion in biological media; and (iii) functionalizing CP surfaces with biomolecules (proteins, peptides, or DNA aptamers) to improve both solubility and targeting ability. Second, embedding CPs in protective matrices such as silica shells, MOFs, hydrogels, or polymeric carriers; incorporating antioxidants or photostable copolymers; and employing optimized excitation conditions with lower-energy light sources are potential solutions for enhancing photostability and operational lifetime of CP fluorescent sensors. Finally, the selectivity and sensitivity of CP fluorescent sensors can be enhanced by (i) incorporating specific recognition sites, such as crown ethers, boronic acids, or metal-binding ligands, into CP structures, and (ii) engineering dual-emission or FRET-based CP systems to enable built-in self-calibration and minimize false positives.

## Figures and Tables

**Table 1:** Summary and comparison of some properties of conjugated polymers and other common materials in the design of fluorescent sensors.

Properties	Conjugated Polymer	Organic Dyes	Quantum Dots	Metal nanoclusters
Amplification efficiency	High (efficient exciton migration, molecular wire effect)	Low (single-fluorophore excitation)	Moderate (can be enhanced with shell coating)	Low
Emission tunability	Highly tunable via backbone engineering or copolymerization	Moderate (limited by chemical structure)	Highly tunable by size and composition	Limited (tuned by core size and ligand environment)
Molar absorptivity	High (broad absorption bands)	Moderate	Very high (broad, strong absorption)	Low to moderate
Quantum yield	High, especially in optimized formulations	High	Moderate to high (improves with passivation)	Moderate
Photostability	High (resistant to photobleaching)	Low (prone to photobleaching)	Very high (excellent photostability)	Moderate to high
Water solubility/dispersibility	Low, often requires encapsulation or surfactant	High	Moderate (requires surface modification)	High
Biocompatibility	Moderate (dependent on functionalization and size)	High	Moderate to high (depends on surface chemistry)	High (depending on ligands used)
Structural precision	Low (polymer chains are polydisperse)	High (defined molecular structure)	High (well-controlled nanocrystal structure)	High (atomically defined clusters)
Reproducibility	Moderate (affected by synthesis variability)	High (synthetically reproducible)	High	High
Aggregation behavior	Prone to ACQ	Stable in dilute solution; aggregation at high concentration	Aggregation risk in salt/bio media	Minimal aggregation; stabilized by ligands
Reaction yield of the synthetic processes	60–90% (highly dependent on monomer solubility and catalyst efficiency) High-molecular-weight formation	70–95% high yield due to well-optimized reactions	40–80% (isolated yield) Yield sensitive to precursor ratios	20–60% (isolated yield) Low yield due to competing nucleation into nanoparticles
Environmental stability	Moderate (prone to photobleaching and degradation under oxygen, moisture, and pH variations)	Poor (susceptible to photobleaching and chemical degradation)	High long-term stability	Low (sensitive to aggregation, surface oxidation, and ligand exchange)

**Table 2:** Summary of recent applications of CP-based fluorescent sensors for the detection of various metal ions.

CPs	Types of Sensors	Metal Ions	LOD	Selectivity	Refs
PPFP-Br *	Bioassay in an aqueous medium	K^+^	1.5 nM	Na^+^, Ca^2+^, Li^+^, Mg^2+^, NH_4_^+^, Cu^2+^	[82]
Poly(fluorene-co-phenylene)	Förster resonance energy transfer (FRET)	K^+^	0.7 nM	Na^+^, Ca^2+^, Hg^2+^, Mg^2+^, Cu^2+^_,_ Fe^2+^	[83]
Cationic polyfluorene	Förster resonance energy transfer (FRET)	K^+^	3 nM	Ag^+^, Ca^2+^, Li^+^, Al^3+^, Cu^2+^_,_ Zn^2+^	[84]
Benzo [1,2-b:4,5-b′]dithiophene	Optical solid state sensor	Na^+^	1 mM	K^+^	[85]
Water-soluble polyfluorene	Aqueous polyfluorene probe	Fe^2+^	46 nM	Cu^+^, Cu^2+^, Co^2+^, Fe^3+^, Na^+^, K^+^, Zn^2+^, Cr^3+^, Ag^+^, Hg^2+^, Cd^2+^, Ni^2+^, Pb^2+^	[86]
Anionic polyfluorene	Aqueous polyfluorene probe	Fe^3+^	0.33 µM	Mg^2+^, Ca^2+^, Mn^2+^, Mn^3+^, Fe^2+^, Al^3+^, Co^2+^, Ni^2+^, Cu^2+^, Zn^2+^, Cd^2+^	[87]
Water-soluble poly(9-aminofluorene)	Fluorescent chemosensor	Fe^3+^	3.7 pM	Mg^2+^, Ca^2+^, Mn^2+^, Fe^2+^, Al^3+^, Co^2+^, Ni^2+^, Cu^2+^, Zn^2+^, Cd^2+^, Hg^2+^, Pb^2+^	[88]
Water-soluble polyfluorenes	Aqueous polyfluorene probe	Fe^3+^	0.8 µM	Mg^2+^, Ca^2+^, Mn^2+^, Fe^2+^, Al^3+^, Co^2+^, Ni^2+^, Cu^2+^, Zn^2+^, Cd^2+^, Hg^2+^, Pb^2+^	[89]
Poly(9-fluorenecarboxylic acid)	Aqueous polyfluorene probe	Fe^3+^	0.611 nM	Pd^2+^, Cu^2+^, Hg^2+^, Ni^2+^, Co^2+^, Zn^2+^, Ba^2+^, Ca^2+^, Mg^2+^, Sn^2+^, Mn^2+^, K^+^, Cr^3+^, Cd^2+^, Al^3+^, Sr^2+^, Pb^2+^	[90]
Poly(5-cyanoindole)	Fluorescent chemosensor	Fe^3+^	16 nM	Cu^2+^, Hg^2+^, Ni^2+^, Co^2+^, Zn^2+^, Ba^2+^, Ca^2+^, Mg^2+^, Sn^2+^, Mn^2+^, K^+^, Cr^3+^, Cd^2+^, Al^3+^, Sr^2+^, Pb^2+^	[91]
Poly(1-amino-5-chloroanthraquinone)	Fluorescent chemosensor	Fe^3+^	0.02 nM	Na^+^, Cu^2+^, Hg^2+^, Ni^2+^, Co^2+^, Zn^2+^, Ba^2+^, Ca^2+^, Mg^2+^, Sn^2+^, Mn^2+^, K^+^, Cr^3+^, Cd^2+^,Al^3+^,Ce^2+^, Pb^2+^	[92]
Poly[p(phenylene ethynylene)-alt-(thienylene ethyn-ylene)]	Förster resonance energy transfer (FRET)	Fe^3+^	0.3 µM	Ag^+^, Cu^2+^, Hg^2+^, Ni^2+^, Co^2+^, Zn^2+^, Ba^2+^, Ca^2+^, Mg^2+^, Yb^3+^, Mn^2+^, Cr^3+^, Cd^2+^, Al^3+^, Ce^2+^, Pb^2+^	[79]
Poly(p-phenyleneethynylene)	Ion-sensing assay	Al^3+^	0.37 μM	Ag^+^, Cu^2+^, Hg^2+^, Co^2+^, Zn^2+^, Ga^3+^, Mg^2+^, Mn^2+^, Cr^3+^, Fe^2+^, Pb^2+^, Fe^3+^	[93]
Anionic polytriazole	Fluorescent chemosensor	Al^3+^	1.1 μM	Na^+^, K^+^, Ag^+^, Cu^2+^, Ni^2+^, Co^2+^, Zn^2+^, Ca^2+^, Mg^2+^, Mn^2+^, Cr^3+^, Fe^3+^, Pb^2+^	[94]
Polythiophene	A colorimetric and fluorescent sensor	Al^3+^	4 μM	Na^+^, K^+^, Li^+^, Cu^2+^, Hg^2+^, Ni^2+^, Co^2+^, Zn^2+^, Ca^2+^, Mg^2+^, Mn^2+^, Cr^3+^, Cd^2+^, Fe^2+^, Pb^2+^, Fe^3+^	[95]
Water-soluble polyfluorenes	Fluorescent chemosensor	Cu^+^ Cu^2+^	0.25 μM 0.25 μM	Na^+^, K^+^, Li^+^, Ag^+^, Ni^2+^, Co^2+^, Zn^2+^, Ca^2+^, Ba^2+^, Cd^2+^	[96]
Amine-functionalized polyfluorene	Fluorescent film sensor	Cu^2+^	5 μM	Na^+^, Cu^2+^, Hg^2+^, Ni^2+^, Co^2+^, Zn^2+^, Ca^2+^, Mg^2+^, Mn^2+^, Ba^2+^, Pb^2+^, Fe^3+^	[97]
Double bond functionalized polyfluorene	Fluorescent optosensor	Cu^2+^	1 nM	Na^+^, K^+^, Hg^2+^, Co^2+^, Zn^2+^, Ca^2+^, Mg^2+^, Mn^2+^, Pb^2+^, Fe^2+^	[98]
Polyfluorene	Fluorescent chemosensor	Cu^2+^	0.5 μM	Na^+^, K^+^, Li^+^, Hg^2+^, Ni^2+^, Zn^2+^, Ca^2+^, Mg^2+^, Mn^2+^, Cr^3+^, Cd^2+^, Fe^2+^, Pb^2+^, Al^3+^, Ba^2+^, Fe^3+^, Ag^+^	[99]
Sulfate-functionalized polyfluorene	Fluorescent chemosensor	Cu^2+^	2.5 µM	Hg^2+^, Ni^2+^, Zn^2+^, Sn^2+^, Mg^2+^, Fe^2+^, Pb^2+^, Al^3+^, Fe^3+^, Ag^+^, Au^3+^, Co^2+^	[100]
Poly(phenylene ethylene)	Fluorescent chemosensor	Cu^2+^	24 nM	Na^+^, K^+^, Li^+^, Hg^2+^, Ni^2+^, Zn^2+^, Ca^2+^, Mg^2+^, Mn^2+^, Cr^6+^, Cd^2+^, Fe^3+^, Pb^2+^, Al^3+^, Ba^2+^, Fe^3+^, Ag^+^, Co^2+^, Pt^2+^, Be^2+^, Cs^+^, As^2+^	[101]
Water-soluble polythiophene	Fluorescent chemosensor	Cu^2+^	10 nM	K^+^, Li^+^, Hg^2+^, Ni^2+^, Zn^2+^, Ca^2+^, Mg^2+^, Mn^2+^, Fe^2+^, Pb^2+^, Ba^2+^, Ag^+^, Co^2+^	[44]
Poly3-[2-(2-methoxy-ethoxy)-ethoxy]-thiophene	Fluorescent chemosensor	Cu^2+^	0.45 μM	Na^+^, Ni^2+^, Zn^2+^, Mg^2+^, Mn^2+^, Cr^3+^, Cd^2+^, Fe^3+^, Sn^4+^, Al^3+^, Ag^+^, Co^2+^	[102]
poly 3-[1-(2-hydrazino-2-oxoethyl)piperidin-4-ylidene]methylthiophene	Fluorescent chemosensor	Cu^2+^ Hg^2+^	20 nM 2 nM	Ni^2+^, Sr^3+^, Cr^3+^, Fe^3+^, Pb^2+^, Al^3+^, Ag^+^, Li^+^, Co^2+^, Cd^2+^, Mn^2+^	[103]
Crosslinked polythiophenes	Fluorescent nano probe	Cu^2+^	10 nM	Na^+^, K^+^, Hg^2+^, Zn^2+^, Ca^2+^, Cd^2+^, Fe^2+^, Pb^2+^, Al^3+^, Fe^3+^, Ag^+^, Co^2+^	[104]
Oligo(1-pyreneboronic acid)	Fluorescent sensor	Cu^2+^	23 pM	Na^+^, K^+^, Hg^2+^, Ni^2+^, Zn^2+^, Ca^2+^, Mg^2+^, Mn^2+^, Cr^3+^, Cd^2+^, Sr^2+^, Pb^2+^, Al^3+^, Ba^2+^, Fe^3+^	[105]
Terpyridyl appended poly(metaphenylene-alt-fluorene)	Turn-off fluorescent probe	Cu^2+^	13–14 μM	Na^+^, K^+^, Hg^2+^, Ni^2+^, Zn^2+^, Ca^2+^, Mg^2+^, Co^2+^, Cr^3+^, Cd^2+^, Sr^2+^, Pb^2+^, Ag^+^, Ba^2+^, Fe^3+^	[106]
2,6-bis(4-bromophenyl)-4-phenylpyridine	Turn-off fluorescent probe	Pd^2+^	1 μM	Pt^4+^, Hg^2+^, Ni^2+^, Zn^2+^, Ca^2+^, Co^2+^, Cd^2+^, Ag^+^, Fe^3+^, Cu^2+^	[107]
Polybenzanthrone	Fluorescent chemosensor	Pd^2+^	0.27 nM	K^+^, Hg^2+^, Ni^2+^, Zn^2+^, Ca^2+^, Mg^2+^, Mn^2+^, Cr^3+^, Cd^2+^, Sr^2+^, Pb^2+^, Al^3+^, Ba^2+^, Fe^2+^, Fe^3+^, Co^2+^	[108]
Pyrene-pyridine oligomer nanorods	Fluorescent nanosensors	Pd^2+^	70 nM	Na^+^, Hg^2+^, Ni^2+^, Zn^2+^, Ca^2+^, Mg^2+^, Mn^2+^, Cr^3+^, Cd^2+^, Pb^2+^, Al^3+^, Ba^2+^, Fe^2+^, Fe^3+^, Co^2+^	[109]
Thiourea-functionalized poly(phenyleneethynylene)	Fluorescent chemosensors	Ag^+^	47.6 μM	Na^+^, K^+^, Zn^2+^, Mn^2+^, Pb^2+^, Co^2+^, Cu^2+^	[110]
Polytriazoles	Fluorescent chemosensors	Ag^+^	0.42 μM	K^+^, Na^+^, Hg^2+^, Ni^2+^, Zn^2+^, Ca^2+^, Mg^2+^, Mn^2+^, Cr^3+^, Cd^2+^, Cu^2+^, Pb^2+^, Ba^2+^, Co^2+^	[111]
2,3-di(pyridin-2-yl)quinoxaline	Colorimetric and fluorescence “turn-off” chemosensor	Ag^+^	0.5 μM	Na^+^, Li^+^, Hg^2+^, Ni^2+^, Zn^2+^, Ca^2+^, Mg^2+^, Cu^2+^, Cr^3+^, Cd^2+^, Sr^2+^, Al^3+^, Ba^2+^, Zr^4+^, Fe^3+^, Co^2+^, La^3+^	[112]
Ethyl 2-(2-(pyridin-2-yl)-1H-benzo[d]imidazol-1-yl)acetate	Fluorescent chemosensor	Ag^+^	0.05 μM	Na^+^, K^+^, Hg^2+^, Ni^2+^, Zn^2+^, Ca^2+^, Mg^2+^, Cu^2+^, Cr^3+^, Cd^2+^, Al^3+^, Ba^2+^, Fe^2+^, Fe^3+^, Co^2+^	[113]
Water-soluble organometallic conjugated polyelectrolyte	Colorimetric and fluorescent sensor	Ag^+^	0.5 μM	Li^+^, Cs^+^, Hg^2+^, Ni^2+^, Zn^2+^, Ca^2+^, Mg^2+^, Cu^2+^, Cd^2+^, Fe^3+^, Co^2+^, Pb^2+^	[114]
1,7-bis((3-formyl-4-hydroxyphenyl) ethynyl)perylene	Off–on fluorescent sensor	Hg^2+^	0.7 μM	Na^+^, K^+^, Ni^2+^, Zn^2+^, Ca^2+^, Ag^+^, Cr^3+^, Cd^2+^, Al^3+^, Fe^3+^, Co^2+^, Pb^2+^	[115]
Poly-p-phenylene	Fluorescence “turn-off/turn-on” and colorimetric sensor	Hg^2+^	2.1 nM	Ni^2+^, Zn^2+^, Mn^2+^, Cu^2+^, Cr^3+^, Cd^2+^, Fe^2+^, Co^2+^	[116]
Cationic polythiophene	Turn-off fluorescent probes	Hg^2+^	1 nM	Li^+^, Ag^+^, Ni^2+^, Zn^2+^, Ca^2+^, Cu^2+^, Cr^3+^, Cd^2+^, Al^3+^, Ba^2+^, Sr^2+^, Pb^2+^, Co^2+^, Mn^2+^	[117]
Poly(benzodithieno-imidazole-alt-carbazole)	Turn-off fluorescent probes	Hg^2+^	0.2 μM	Na^+^, K^+^, Pb^2+^, Ni^2+^, Zn^2+^, Ca^2+^, Mg^2+^, Cu^2+^, Cr^3+^, Cd^2+^, Al^3+^, Ba^2+^, Sr^2+^, Fe^3+^, Co^2+^, Ag^+^	[118]
Polydiacetylene	Dual colorimetric and fluorometric sensor	Pb^2+^	3.8 μM	Li^+^, K^+^, Ni^2+^, Zn^2+^, Ca^2+^, Mg^2+^, Cs^+^, Cr^3+^, Cd^2+^, Al^3+^, Mn^2+^, Co^2+^	[119]
Polydiacetylene vesicles	Colorimetric and fluorescent sensor	Pb^2+^	5 μM	Na^+^, K^+^, Li^+^, Ni^2+^, Zn^2+^, Ca^2+^, Mg^2+^, Cu^2+^, Hg^2+^, Fe^2+^, Co^2+^, Cd^2+^	[120]
Polydiacetylene	Fluorescent and colorimetric sensors	Pb^2+^	1 μM	Na^+^, Li^+^, Ni^2+^, Zn^2+^, Mg^2+^, Cu^2+^, Cr^3+^, Cd^2+^, Al^3+^, Fe^3+^, Co^2+^, Ag^+^, Hg^2+^, Mn^2+^	[121]
Polydiacetylene liposome	Fluorescent and colorimetric sensors	Pb^2+^	3 μM	Na^+^, K^+^, Mn^2+^, Ca^2+^, Mg^2+^, Cu^2+^, Cd^2+^, Ba^2+^, Fe^3+^, Co^2+^, Ag^+^, Hg^2+^	[122]
Polydiacetylene liposome	Colorimetric and fluorescent sensor	Pb^2+^	0.1 mM	Ni^2+^, Fe^2+^, Ca^2+^, Mg^2+^, Cu^2+^, Cd^2+^, Co^2+^, Hg^2+^	[123]
Polydiacetylene dots	Dual colorimetric and fluorescent sensor	Pb^2+^	0.5 μM	Ni^2+^, Fe^2+^, Ca^2+^, As^3+^, Mn^2+^, Cd^2+^, Co^2+^, Cu^2+^	[124]
Water-soluble cationic polythiophene	Fluorescent sensor	Pb^2+^	6 nM	Na^+^, K^+^, Li^+^, Ca^2+^, Mg^2+^, Cu^2+^, Cd^2+^, Ba^2+^, Fe^2+^, Co^2+^, Zn^2+^, Hg^2+^, Ni^2+^	[125]
Benzo[a]imidazo [5,1,2-cd] fluorophores	Bicolor fluorescent sensor	Ba^2+^	0.059 μM	Na^+^, K^+^, Ca^2+^, Mg^2+^, Sr^2+^	[80]
Benzo [4,5]imidazo [2,1,b] quinones	Bicolor fluorescent sensor	Cu^2+^	74.4 nM	Na^+^, K^+^, Sr^2+^, Cr^3+^, Cd^2+^, Ba^2+^, Fe^3+^, Co^2+^, Zn^2+^, Al^3+^, Ni^2+^	[81]

* PPFP-Br: Poly[(9,9′-bis(4-(6-N,N,N-trimethylammoniumhexyloxy)phenyl)fluorene-2,7-diyl)-alt-1,4-phenylene dibromide].

**Table 4:** Summary of recent applications of CP-based fluorescent biosensors for the detection of various pathogens.

CPs	Types of Sensors	Pathogen	LOD	Refs
PEDOT	Quartz crystal microbalance	H1N1	0.12 HAU	[172]
Poly(l-lysine) (PLL)/poly(acrylic acid) (PAA)	Metal-enhanced fluorescence	*E. coli*	5 × 10^5^ CFU	[171]
Cationic polythiophene derivative	Fluorescence biosensor	*S. aureus* *E. coli* *C. albicans*	1.2 × 10^8^ CFU/mL 1.0 × 10^8^ CFU/mL 4 × 10^7^ CFU/mL	[173]
Cationic porphyrin	Förster resonance energy transfer	*E. coli*	0.1 × 10^6^ CFU/mL	[174]
2,6-Sialyllactose-DCM	Förster resonance energy transfer	H3N2 H7N9 H10N8	256 HAU	[175]
Cationic poly(fluorene-co-phenylene)	Fluorescence biosensor	*B. subtilis* *E. faecalis* *S. aureus* *E. coli* *P. aeruginosa* *C. albicans* *S. cerecisiae*	-	[176]
Cationic poly(phenylene vinylene) derivative	Fluorescence biosensor	*B. subtilis* *E. coli* *C. albicans*	OD 600 = 1.0 OD 600 = 1.0 OD 600 = 2.0	[177]
PFDBT-BIMEG *	Förster resonance energy transfer	*S. aureus* *E. coli* *C. albicans*	OD600 = 0.5	[178]
Poly(fluorene-co-phenylene)	Fluorescence biosensor	*S. aureus*	OD600 = 2.0	[179]
Polythiophene	Fluorescence biosensor	*S. aureus* *S. epidermidis*	OD600 = 1.0	[180]
Cationic conjugated glycopolymer	Förster resonance energy transfer	*E. coli*	OD600 = 0.025	[181]
Poly(3,4- propylenedioxythiophen-alt-3,4-ethylenedioxythiophene) copolymer (PPE)	Colorimetric and fluorescence biosensor	*S. aureus* *E. coli*	OD600 = 0.25	[182]

* PFDBT-BIMEG: poly[(9,9-bis6′-[N-(triethylene glycol methyl ether)-di(1H-imidazolium)methane]hexyl-2,7-fluorene)-co-4,7-di-2-thienyl-2,1,3-benzothiadiazole] tetrabromide.

## Data Availability

No new data were created or analyzed in this study.
